# Organic-nanoclay composite materials as removal agents for environmental decontamination

**DOI:** 10.1039/c9ra08230a

**Published:** 2019-12-06

**Authors:** Giuseppe Cavallaro, Giuseppe Lazzara, Elvira Rozhina, Svetlana Konnova, Marina Kryuchkova, Nail Khaertdinov, Rawil Fakhrullin

**Affiliations:** University of Palermo, Department of Physics and Chemistry viale delle Scienze pad. 17 Palermo 90128 Italy giuseppe.lazzara@unipa.it kazanbio@gmail.com; Institute of Fundamental Biology and Medicine, Kazan Federal University Kreml uramı 18 Kazan Republic of Tatarstan 420008 Russian Federation

## Abstract

Here we overview the recent advances in the fabrication of sustainable composite nanomaterials with decontamination capacity towards inorganic and organic pollutants. In this regards, we present the development of hybrid systems based on clay nanoparticles with different shapes (such as kaolinite nanosheets and halloysite nanotubes) and organic molecules (biopolymers, surfactants, cucurbituril) as efficient removal agents for both aliphatic and aromatic hydrocarbons. Due to their high specific surface area, clay nanoparticles have been successfully employed as fillers for composite membranes with excellent filtration capacity. The preparation of composite gel beads based on biopolymers (alginate and pectin) and halloysite nanotubes has been discussed and their adsorption capacities towards both heavy metals and organic dyes have been highlighted. We describe the successful preparation of kaolinite/graphene composites as well as tubular inorganic micelles obtained by the select functionalization of the halloysite cavity with anionic surfactants. Finally, recent research on Pickering emulsions (for oil spill remediation) and bioremediation technologies has been discussed.

## Abstract 2

Here we overview the recent advances in the fabrication of sustainable composite nanomaterials with decontamination capacity towards inorganic and organic pollutants.

## Graphical abstract

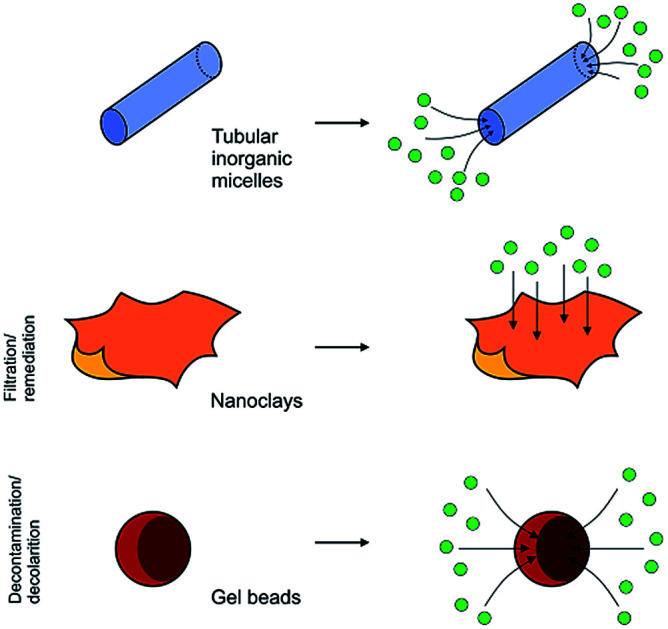

## 1. Introduction

Nowadays, environmental pollution is one of the biggest world problems. Contaminants have been present in the environment since time immemorial: including elements of volcanic dust, comets and cosmic dust, which account for about 100 tons of organic dust per day.^1^ Currently, the spectrum of pollutants has expanded significantly including heavy metals,^2^ hydrocarbons (aliphatic, aromatic and polycyclic aromatic hydrocarbons),^3^ BTEX (benzene, toluene, ethylbenzene and xylenes),^4^ chlorinated hydrocarbons,^5^ trichloroethylene (TCE) and perchloroethylene,^6^ nitroaromatic compounds,^7^ organophosphorus compounds^8^ and pesticides.^9^ Generally, pollutants enter wastewater and then are present in rain, fog and snow.^10^ Oil pollution is directly related to human factors, such as the deliberate discharge of waste.^11^

Nanotechnology products are considered as very effective tools for environmental clean-up. The use of many types of nanoparticles and methods for cleaning natural resources and improving the quality of life of the population is widely described. For example, nanofilter membranes are widely used to remove dissolved salts and micropollutants, as well as water softening and wastewater treatment.^12^ The use of nanomaterials for the treatment of water resources and soils has been demonstrated for chitosan and silver nanoparticles^13,14^ as well as for carbon nanomaterials.^15^ The use of zero-valence iron as a reducing agent for cleaning contaminated sites from polychlorinated biphenyls, pesticides, herbicides, aromatic hydrocarbons and metals has been reported.^16–19^ Nanomaterials-based remediation methods include the use of nanomaterials for detoxification and transformation of pollutants. In this regards, titanium oxide can be used in the photo-oxidation of organic pollutants.^20^ Biodegradation, sorption, hydrolysis, photolysis and microfiltration, UV irradiation represent alternative strategies for the pollutants removal.^21,22^

Recently, various types of nanoclays have become important targets for applications in environmental industries and bioremediation.^23^ For example, clay nanoparticles may absorb various pollutants, including organic (atrazine, trifluralin, parathion and malathion) and inorganic compounds (for example, metals: copper, zinc, cadmium and mercury, *etc.*) from soil and wastewater. In addition, industrial and biomedical use of nanoclays is increasing: planar nanoclays bentonite,^24^ montmorillonite,^25^ kaolin^26^ and tubular mineral halloysite^27^ have been used as nanoscale fillers for the manufacture of polymer composites,^28^ anti-corrosion and flame retardant coatings.^29,30^ The impressive effect of nanoclay particles to improve the structural^31^ and functional properties of biomaterials, along with their availability and low-cost production, suggests the nanoclay's use will constantly increase^32^ Sorbents based on clay minerals have unique properties, such as high specific surface area, reusability, low cost, and ubiquity in the natural environment.^33^ There are reports on the use of bentonite for the sorption of amoxicillin antibiotic from liquid suspensions,^34^ and the adsorption of trimethoprim using montmorillonite clay.^35^ In addition, halloysite nanotubes are considered as an adsorbent for purifying water, for example, from heavy metals,^36^ dyes^37^ and aromatic pollutants.^38–40^ Recent studies provide an opportunity to improve clay minerals, allowing to increase their sorption capacity (from 67.0% to 98.9%) and selectivity for specific metals, thereby opening up a new sphere for their use.^41^ Researchers proposed clay/Fe_3_O_4_ composites as adsorbents to remove heavy metals and dye molecules from modelled wastewater through magnetic separation.^42–44^ Analysis of recent literature sources has shown that textile wastewaters are considered to be the most polluting among all other industrial effluents due to their complex composition^45^ and the possibility of using natural minerals to clean them should be carefully investigated. Moreover, there are several advantages concerning the possibility to recover and to reuse clays after the decontamination process. In the oil recovery, clays can be separated from the emulsion by simple centrifugation or sedimentation process being of larger density compared to water and micron-sized particles, of course this is not the case for conventional surfactants that can be hardly separated from the emulsion. As concerns metal adsorption, the ion release can be achieved after purification by controlling pH and/or ionic strength that typically affect the metal–clay interactions.

Thus, the achievements of nanotechnology can be used for bioremediation, directly or indirectly, for the treatment of surface water, groundwater and wastewater polluted with toxic metal ions, organic and inorganic solutes and microorganisms. Due to their unique activity, nano-sized particles increase the efficiency of absorption of pollutants and are relatively inexpensive compared to traditional sedimentation and filtration methods.

## 2. Water filtration by nanoclays

### 2.1 Comparison between halloysite and kaolinite

**Fig. 1:**
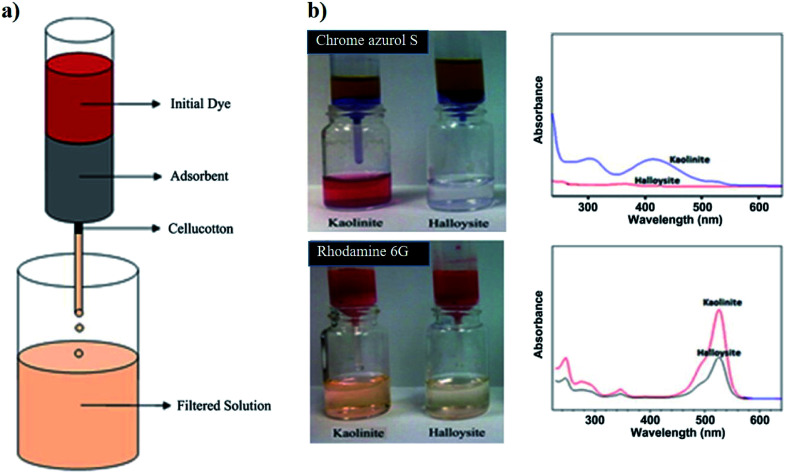
(a) Sketch of the water filter experiment. (b) Demonstration of the water filters prepared by 1 g of halloysite and kaolinite clays tested on Chrome azurol S and Rhodamine 6G solutions with 300 mg dm^−3^ concentration. UV spectra of the filtered water samples are demonstrated on right for clear presentation of the unfiltered dye. Reproduced from ref. 46 with permission from the Elsevier, copyright 2013.

Clay nanoparticles represent suitable adsorbent materials for the water filtration as shown in several research articles^46,47^ and reviews.^48^ In this regards, halloysite was successfully used for efficient water filtration as demonstrated in adsorption studies using cationic Rhodamine 6G and anionic Chrome azurol S as cationic and anionic dyes, respectively.^46^Fig. 1a sketches the filtration tests. Halloysite exhibited a higher removal capacity towards both dyes (Fig. 1b) due to its larger specific surface area (40.2 m^2^ g^−1^) if compared to that of kaolinite (21.3 m^2^ g^−1^). Both nanoclay based filters can be regenerated up to five times by burning the adsorbed dyes.

### 2.2 Water filtration by electrospun membranes based on polyacrylonitrile and halloysite

**Fig. 2:**
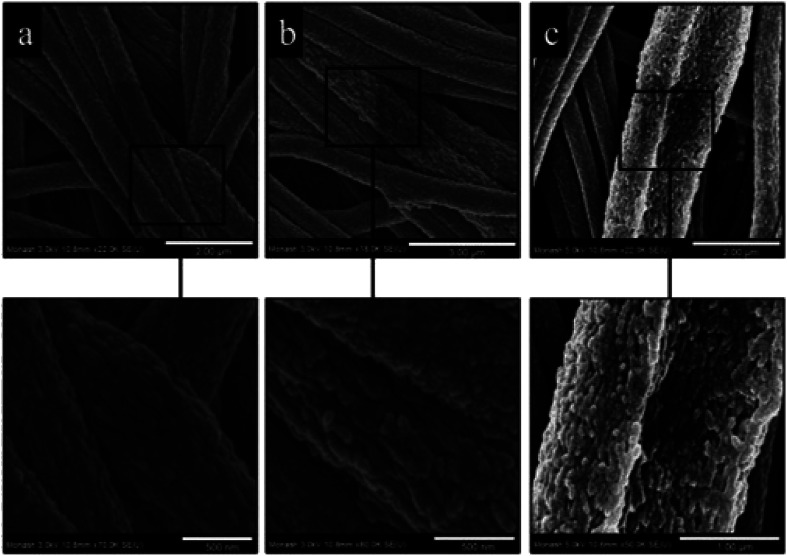
High magnification FE-SEM micrographs of electrospun PAN nanofibers containing the following: (a) 0% w/w HNTs; (b) 1% w/w HNTs; (c) 3% w/w HNTs. Reproduced from ref. 47 with permission from the American Chemical Society, copyright 2015.

The addition of halloysite nanotubes into polyacrylonitrile (PAN) revealed as an efficient strategy to obtain nanofibrous membranes with excellent water filtration capacity.^47^ As displayed in Fig. 2, the nanotubes are well dispersed within the PAN fibrous matrix.

A significant enhancement of the oil removal efficiency was induced by the addition of halloysite into the PAN membrane. The removal efficiency of the membrane based on pure PAN was 4.7%, while the PAN/halloysite (99 : 1) and PAN/halloysite (97 : 3) composites showed adsorption capacities of 10.6 and 31.1%, respectively. These results highlight that the addition of small amounts of nanotubes into the polymer induce significant improvement in the water filtration capacity of PAN based membrane. Moreover, PAN reinforced with 3 wt% of halloysite showed an increase by 740% for the heavy metal adsorption. Besides the enhanced adsorption capacity, the filling with halloysite caused relevant improvements of the thermal stability and mechanical properties (in terms of elongation and tensile strength) of PAN membranes.

## 3. Hybrid gel beads for water decontamination

### 3.1 Alginate/pectin gel beads for removal of heavy metals

**Fig. 3:**
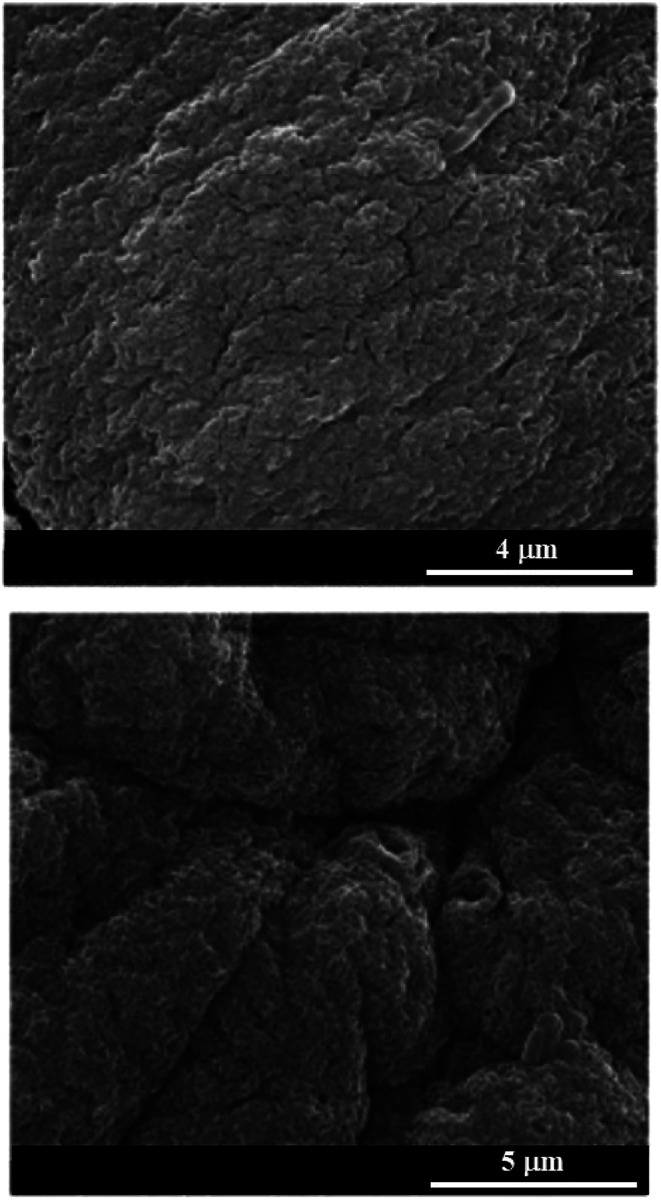
Scanning electron microscopy images of alginate/pectate (1 : 2) beads before and after the sorption of both cadmium(ii) and copper(ii) ions. Reproduced from ref. 49 with permission from the Elsevier, copyright 2013.

The combination of negatively charged biopolymers (alginate and pectate) was explored to fabricate hybrid gel beads to obtain novel heavy metals adsorbents.^49^ The composite gel beads were prepared through the dropping technique. Mixed gel beads exhibited lower density if compared with that of alginate based gel beads. On the other hand, gel beads containing pectate demonstrated the improved mechanical resistance compared to that of pure alginate beads as a consequence of the more compact structure. Adsorption tests demonstrated that the alginate/pectate system in gel phase can be considered a suitable and environmentally safe material for the removal of cadmium(ii) and copper(ii) from aqueous solution. Fig. 3 shows SEM images of alginate/pectate (1 : 2) beads before and after the sorption of both cadmium(ii) and copper(ii) ions. As a general result, the surface of the beads was not significantly altered by the adsorption of the metal ions.

### 3.2 Alginate/halloysite gel beads for water decolouration

**Fig. 4:**
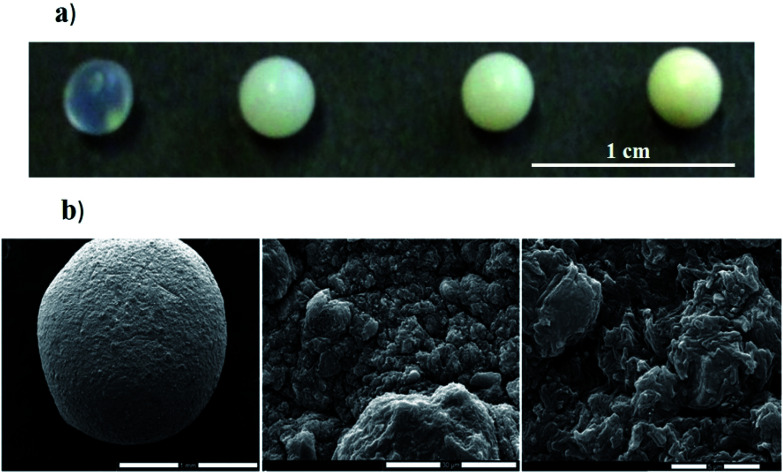
(a) Photographs of hybrid gel beads at variable halloysite content. (b) Scanning electron microscopy images of gel beads loaded with HNTs. Reproduced from ref. 50 with permission from the Elsevier, copyright 2013.

Composite gel beads based on alginate and halloysite nanotubes were prepared by the dropping technique.^50^ As shown in Fig. 4a, the gel beads size was not significantly affected by the halloysite fraction, while their transparency is being reduced upon the addition of the nanotubes.

As shown in SEM images (Fig. 4b), dried hybrid beads possesses a rough surface with pores in the micrometre range that is similar to that observed in the absence of HNTs.^49^ Water decolouration performances of the alginate/halloysite beads were tested by using Crystal Violet (CV) as dye. The adsorption isotherm of CV onto alginate gel beads shows an interesting dye extraction ability from the aqueous phase. Table 1 shows that the presence of halloysite in the gel beads improves the adsorption capacity (expressed in terms of maximum adsorption capacity (*q*_max_)). Regarding the adsorption constant (*K*), it was detected that the dye affinity towards hybrid beads is greater than that towards the pure alginate.

**Table 1:** Experimental parameters for the CV adsorption onto alginate/HNTs gel beads

Halloysite content/wt%	*K*/dm^3^ mol^−1^	*q*_m_/mg g^−1^
0	(39.3 ± 0.8) × 10^4^	8.5 ± 1.0
33.2	(42.4 ± 0.6) × 10^4^	12.7 ± 1.0
48.4	(41.7 ± 0.6) × 10^4^	15.5 ± 1.1
60.2	(42.9 ± 1.7) × 10^4^	17 ± 3

Gel beads based on alginate and halloysite were effective in the removal of methylene blue from aqueous phase. Specifically, the removal efficiency was above 90%.^51^ Similarly to alginate/halloysite systems, the combination of clay nanotubes with chitosan allowed to obtain composite hydrogel with excellent adsorption capacity towards different dyes (such as methylene blue and malachite green) solubilized in water.^52^ Chitosan/halloysite gel beads were prepared by using the dropping and pH-precipitation technique. The addition of halloysite significantly improved the adsorption capacities (72.60 and 276.9 mg g^−1^ for methylene blue and malachite green, respectively) of chitosan based gel beads.^53^

## 4. Kaolinite/graphene composites for remediation

In a recent paper, planar kaolin nanoclay was demonstrated to significantly reduce the toxicity of graphene oxide nanoplates in the aqueous phase. The authors conducted studies on the ciliate *Paramecium caudatum* to identify the effect of different concentrations of the planar kaolin nanoclay and graphene oxide nanoplates. The nanoparticles used by the authors had a similar size distribution (hydrodynamic diameters are approximately 1.9–2.2 μm), and also both had negative zeta potentials (−47 mV for graphene oxide and −22 mV for kaolin). The introduction effect of graphene and graphene oxide into the environment, separately and in combination, was evaluated on the following physiological parameters of ciliates: chemotaxis, galvanotaxis, growth rate, DNA complexation, phagocytic activity. The authors emphasize that the toxicity of graphene oxide nanoplates which coagulated with kaolin was reduced without the removal of nano-conglomerates from the environment. At the same time, graphene oxide plates were highly toxic for *P. caudatum* in the concentration range from 500.0 to 1000.0 μg ml^−1^. The GO concentration at 1000.0 μg ml^−1^ reduced the survival rate of ciliates and 55% of them died after 24 h. At the same time, the addition of graphene oxide plates to the incubation environment with planar kaolin nanoclay and incubation for 24 hours significantly reduced the negative effect of graphene oxide. For example, in the presence of kaolin with adsorbed graphene oxide at a concentration of 1000.0 μg ml^−1^, only 7% of the protists died after 24 h. That is, after the adsorption of graphene oxide by kaolin, the toxicity of graphene oxide in an aqueous environment decreases by approximately 7.8 times.

**Fig. 5:**
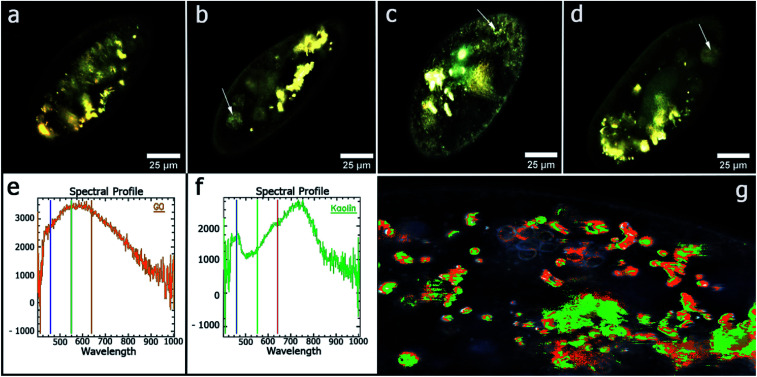
Microscopy images of *P. caudatum* cells, obtained using dark-field microscopy: (a) untreated cells; (b) cells treated with kaolin (100 μg ml^−1^) (food vacuoles are clearly seen); (c) cells treated with graphene oxide (100 μg ml^−1^) (no digestive vacuoles seen); (d) treated with a mixture of kaolin and graphene oxide (100 μg ml^−1^) (digestive vacuoles are observed); (e) the reflected light spectrum of pure graphene oxide; (f) spectrum of the reflected light of pure kaolin; (g) hyperspectral map of distribution of kaolin and graphene oxide in the *P. caudatum* cell. Reproduced from ref. 56 with permission from the American Chemical Society, copyright 2018.

Among the methods used to study the distribution of graphene oxide and kaolin in *P. caudatum* cells, dark field microscopy with hyperspectral mapping was applied.^54^ Spectral libraries of kaolin and graphene oxide were collected, which were subsequently used to analyse the distribution of both types of nanoparticles in living cells of *P. caudatum* (Fig. 5). Graphene oxide nanoplates and planar kaolin nanoclay nanoparticles were diffusely distributed in the cytoplasm, in the digestive vacuoles and in the macronucleus. As a result, the authors showed that kaolin significantly reduces the toxic effect of graphene oxide associated with the membrane. It is assumed that planar kaolin nanoclay interacts with graphene oxide plates; it leads to a weakening of the chemical properties of the latter. The mechanisms of nanotoxicity of graphene-related materials have not yet been fully studied. Since both types of nanomaterials have negative zeta potentials, the electrostatic interaction between the studying particles is unlikely to be effective, as was previously demonstrated for heteroaggregation of graphene oxide with montmorillonite, kaolinite and goethite.^55^ However, atomic force microscopy was successfully used to demonstrate that planar kaolin nanoparticles strongly aggregate with graphene oxide plates.^56^ Thus, for the first time, the authors of this work^56^ describe a significant reduction in the toxicity of graphene oxide plates using planar kaolin nanoclay in an aqueous medium. Using atomic force microscopy, it has been shown that kaolin coagulates with graphene oxide in water, forming relatively large conglomerates, which reduces the side negative effects of graphene oxide on *P. caudatum* ciliates, which potentially has high practical significance in the field of graphene materials.

## 5. Nanosponges based on halloysite and cucurbituril

**Fig. 6:**
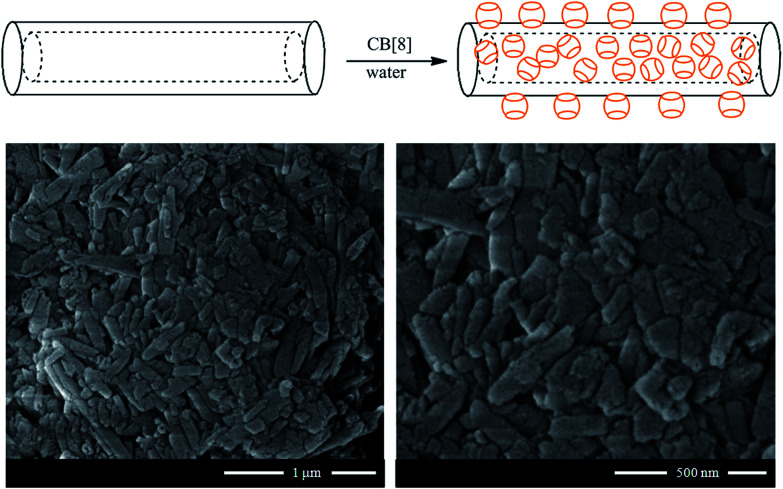
(Top) Schematic representation of the preparation of CB/HNT supramolecular complex; (bottom) SEM images of CB[8]/HNT nanosponge. Reproduced from ref. 57 with permission from the John Wiley and Sons, copyright 2016.

Nanosponges composed of halloysite and cucurbituril molecules were prepared in order to fabricate biocompatible materials with excellent adsorption capacity towards hydrocarbons. Specifically, cucurbit[8]uril (CB[8])^57^ and cucurbit[6]uril^58^ were employed for the functionalization of halloysite nanotubes. As sketched in Fig. 6, the supramolecular complex between halloysite and CB molecule was prepared by mixing halloysite with a saturated solution of CB in water, which has low viscosity. The CB/HNT dispersions was stirred and kept under vacuum for 3–5 min. Then the vacuum was broken, solution entered into lumen and loaded compound condensates within the tube. This was repeated 2–3 times to increase the loading efficiency. After loading, tubes were washed several time with water in order to remove the CB did not interact and dried under vacuum at 70 °C (Fig. 6).

The procedure described allowed generating modified HNT with a relevant functionalization degree. Based on thermogravimetric analysis, it was estimated that the amounts of CB[8] and CB[6] adsorbed onto the halloysite surfaces were 25 and 39 wt%, respectively. As shown in Fig. 6, the adsorption of CB molecules did not affect the peculiar tubular morphology of halloysite.

The supramolecular structures were tested for entrapping toluene both in the vapour and liquid phase. As concerns the adsorption experiments in vapour phase (Table 2), the hybrid nanomaterial exhibited a strong enhancement of capability in capturing toluene gas compared to the pristine HNT. At a given time, the adsorbed amount is larger for the nanohybrids; for instance, after 2 h, the toluene amount captured by the nanohybrid is *ca.* 50 times larger than that of pristine HNT.

**Table 2:** Parameters for the capture of toluene in vapour phase by HNT and HNT/CB[8] at 25 °C^a^

Time (h)	HNT/CB[8]	HNT
	*W*_oil_	*W*_oil_
2	18.0 ± 0.2	0.37 ± 0.01
3	36.9 ± 0.4	1.38 ± 0.04
20	38.6 ± 0.4	10.7 ± 0.1

a *W*_oil_, mg toluene/g adsorbent.

Adsorption experiments in liquid phase evidenced that the presence of CB[8] onto the nanoclay surfaces significantly improves the HNT removal ability towards toluene dissolved in water. Spectroscopy measurements provided the partition coefficient of toluene (*P*) in aqueous dispersions of pristine and modified HNT. It was detected that *P* for CB[8]/HNT is three times larger with respect of that calculated for pristine HNT. Particularly, *P* values are 215 ± 8 and 72 ± 5 for HNT/CB[8] and HNT, respectively. These results indicate that the modified nanotubes possess a larger affinity toward toluene because of the presence of hydrophobic domains (composed by CB[8] molecules) on HNT surfaces. On the other hand, the lower adsorption ability of the pristine nanotubes correlates well with the HNT hydrophilic nature. The mechanism of interactions between HNT/CB[8] and hydrocarbons was clearly confirmed by fluorescence spectroscopy experiments, which were carried out by using pyrene as a fluorescent probe.

## 6. Tubular inorganic micelles: halloysite/surfactant hybrids

**Fig. 7:**
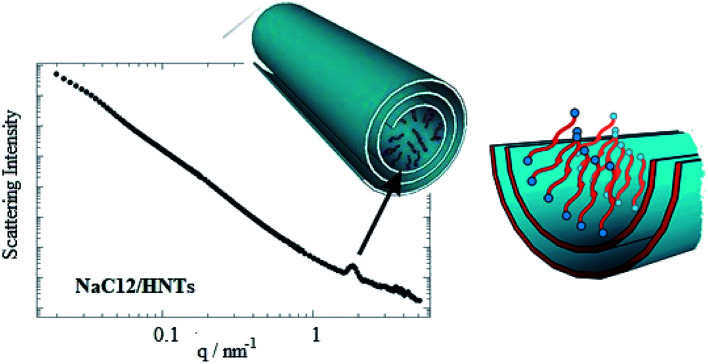
SANS curve for dispersion of NaC12/HNTs in D_2_O. A scheme demonstrating the surfactant organization within the halloysite lumen. Reproduced from ref. 60 with permission from the American Chemical Society, copyright 2016.

Selective modification of halloysite positive inner surface by anionic surfactants allowed fabricating hybrid nanotubes with a hydrophobic cavity and a hydrophilic shell. Sodium alkanoates,^38,59,60^ sodium perfluoroalkanoates^61,62^ and sodium dodecylsulfate^60^ were employed as anionic surfactants. The preparation of the hybrids was conducted by mixing HNT powders with saturated aqueous solutions of the surfactants (sodium dodecanoate NaC12, sodium tetradecanoate NaC14, sodium dodecylsulphate NaDS and sodium perfluoroalkanoates). The functionalized HNTs can be considered as removal agents for decontamination purposes due to their capacity to entrap aliphatic and aromatic hydrocarbons inside their hydrophobically modified lumen.^38^ Both the head polar group and the length of the hydrocarbon chain affect the surfactant loading. According to the HNTs sizes, the maximum loading value expected from the cavity is *ca.* 10 vol%,^63^ which is almost reached for NaC12/HNTs and NaC14/HNTs hybrids. Namely, loading of the HNTs lumen with the formation of surfactant complex structures was detected for sodium alkanoates with longer alkyl chain. The lower amounts of NaDS and sodium pefluoroalkanoates entrapped into the HNTs cavity are in agreement with a surfactant monolayer adsorption by taking into account the average specific area of the halloysite inner surface (6.9 m^2^ g^−1^)^38^ and the occupied area of the carboxylate and sulphate head groups. SANS data (Fig. 7) confirmed that the structural organization of the surfactants adsorbed onto the HNTs lumen depends on their polar head group.^60^

**Fig. 8:**
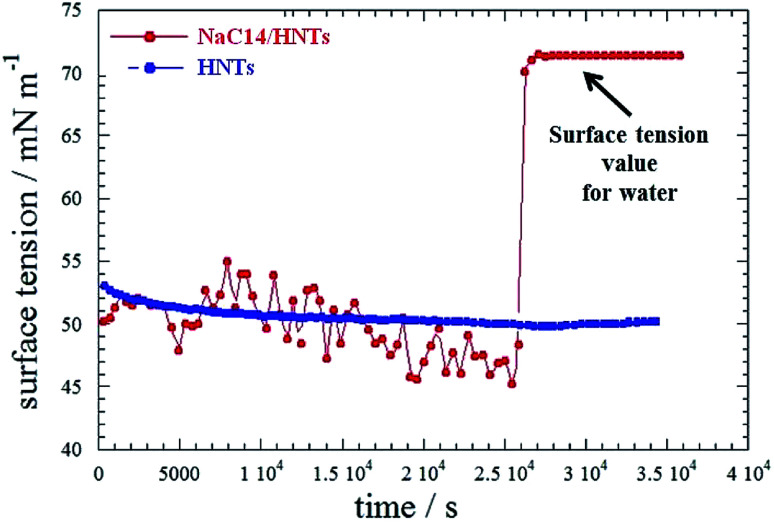
Surface tension as a function of time for aqueous HNT and HNT/NaC14 dispersions in the presence of an *n*-decane film. Reproduced from ref. 38 with permission from the American Chemical Society, copyright 2014.

In particular, SANS curves of NaC12/HNTs showed a peak at *q* (magnitude of the scattering vector) = 1.79 nm^−1^, which could be correlated to the formation of multilayers structures or cylindrical packing of surfactant within the HNT lumen. The formation of complex surfactant structures within the halloysite lumen improved the solubilisation ability of halloysite towards hydrocarbons.^38^ As for the pure halloysite, nanotubes modified with NaC12 and NaC14/exhibited an enhancement of the toluene removal efficiency of *ca.* 9 and 18%, respectively. Moreover, the surfactant/halloysite composites were efficient in the removal of liquid *n*-decane as a consequence of the hydrophobization of the lumen.^38^ Thermogravimetric volatilization experiments on *n*-decane equilibrated with halloysite powders highlighted the confinement of the aliphatic hydrocarbon within the lumen of NaC12/HNTs. The capability to remove an oil film at the water/air interface by using surfactant/HNTs systems was proved by time-resolved surface tension measurements (Fig. 8). This property was not observed for pure halloysite because of its hydrophilic nature.

## 7. Pickering emulsions for oil spill remediation

The formation of a Pickering emulsion (oil-in-water) stabilized by nano- or/and micro-particles can be considered as an alternative approach to increasing the surface area of the oil. This is relevant, for example, in terms of incident happened in the Gulf of Mexico.^64^ Among the particles that can form the boundary between oil and water are described silica, latex, clay, even bacterial cells^65,66^ and halloysite clay nanotubes are also suitable candidates for such Pickering emulsions formation. The formation of emulsion with crude oil was shown previously.^67–69^

Native halloysite nanotubes have an electrical zeta potential of about −30 mV and a contact angle of about (13 ± 2)°, but it is possible to modify the surface properties of the halloysite mineral nanoclay by its silanization and increasing the contact angle to (99 ± 30)°. Hydrophobization of the inner surface of HNTs with silanes increases the stability of the emulsion.^69^ It has been shown that the loading of widely used surfactants (Span 80, lecithin, *etc.*) into the lumen of nanotubes of halloysite significantly increases the dispersibility of crude oil.^70^

Alkanes constitute the largest part of crude oil by weight, that is why the biodegradation of this fraction is the most crucial when removing crude oil from the environment. In this study, the authors initially describe the use of native and silane-modified halloysite nanotubes for the emulsification of the *n*-hexadecane model alkane and demonstrate that this approach works on Macondo crude oil. Moreover, the authors show that the use of the natural mineral halloysite attracts alkane-decomposing bacteria *A. borkumensis* and stimulates the viability of *A. borkumensis* cultivated with hexadecane or crude oil acting as the sole source of carbon.

The authors estimated bacteria viability by evaluating the growth of *A. borkumensis* in marine broth supplemented with *n*-hexadecane (or crude oil) as the sole source carbon in the presence of the native mineral halloysite and ODTMS modified halloysite (HNT 99°). Using the growth curves, the growth rate and the doubling time of the microorganisms were calculated. The doubling time (generation) is the time required for the cells to double their numbers. The average growth rate of bacteria in the control sample was 0.3 per hour, which corresponds to a generation time of 2.3 hours, while the growth rate of bacteria in an environment with nanomaterials of 0.25, 0.5 and 1 wt% was 0.28 hours, which corresponds to a generation time of 2.5 hours. These data are consistent with the growth rates of *A. borkumensis* on alkanes of different lengths (the growth rate of bacterial *n*-hexadecane is 0.3 hours).^71^

Thus, the addition of halloysite aluminosilicate slightly increases the generation time. This is probably due to the fact that bacteria require more time for transition into the medium containing colloidal particles. We did not monitor significant difference in growth rate and generation time in cultures supplemented with hydrophobised halloysite ODTMS (contact angle 99°). Then, the effect of the halloysite mineral on the metabolic activity of *A. borkumensis* was investigated. The metabolic activity of bacteria plays an important role in assessing the ability of cells to consume and convert nutrients. This is not only an indicator of cell viability, but also an activity that is relevant for the survival of bacterial enzymes, such as dehydrogenase.

To assess the metabolic activity of bacteria in the presence of halloysite nanotubes the authors used *in vitro* method for assessing the activity of dehydrogenase enzymes that reduced non-fluorescent blue resazurin to fluorescent pink resorufin.^72^ It was made to demonstrate the kinetics of the production of resorufin by bacteria in the presence of 0.2 mg ml^−1^ halloysite for 40 hours. The growth of *A. borkumensis* in both cases was supplemented with 1% hexadecane. The analyte was filtered before analysis to eliminate interference in resorufin. The supplement of native mineral halloysite significantly increased the rate of resorufin formation, which indicates an increase in the activity of bacterial enzymes. Efficiency of metabolism causes a spurt of enzyme activity after 24 hours of incubation with halloysite, when the rate of production of resorufin is 2–4 times higher than the proliferation of bacteria at the oil/water interface. We can see that the metabolism of *A. borkumensis* increases in the medium with the supplement of halloysite. *A. borkumensis* is a common marine bacterium that degrades the aliphatic portion of crude oil. Therefore, we used *n*-hexadecane as the primary model of an aliphatic source of carbon. After 2–3 days of growth in a liquid culture medium the formation of white flakes, floating in the upper part of the culture medium, is observed. A microscopic observation of these flakes revealed a biofilm containing a microemulsion of an oil droplet with a diameter of 10–50 μm. Examination of the sample under an optical microscope with an immersion objective showed that oil droplets contain short (1.5–2.5 μm) rod-shaped bacterial cells.

Then, the authors formed *n*-hexadecane emulsions in the marine broth containing ODTMS-hydrophobised halloysite nanotubes (contact angle of 99°), after inoculation with *A. borkumensis* to a final concentration of 10^7^ cells per ml. After 5–7 days the emulsions containing bacteria were observed. It is seen that the bacteria lengthen and form a dense patterned biofilm on the surface of the oil droplets. Biofilm is formed as a result of the growth of bacteria between halloysite aggregates. To test the ability of *A. borkumensis* to proliferate on the surface of crude oil emulsions as compared with detergent-based surfaces, oil emulsions with ablation of artificial organic dispersants, as well as halloysite, were stabilized. The mixture of dispersants consisted of 48% non-ionic and 35% anionic substances similar in composition to one used during the oil spill in Horizon.^73^ Subsequently, the mixture was blended with similar compounds, such as corexit, which were used during the spill.

**Fig. 9:**
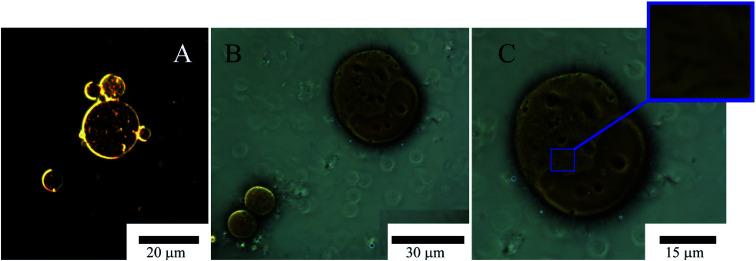
Optical microscopy images of Pickering emulsion, emulsion of petroleum formed exclusively by halloysite nanotubes (A) and with *A. borkumensis* (B and C) after 3 days incubation in marine broth at 30 °C, showing general morphology under low magnification and view of a bacteria *A. borkumensis* inside it (C).

Microscopic studies of these emulsions for one and three days after the supplement of bacteria showed that several bacterial cells were attached to the oil/water interface in emulsions obtained with the dispersing mixture and (Fig. 9) one to three days after inoculation. In samples containing native halloysite, attaching of cells to emulsions occurred at the same time (Fig. 9). We did not observe further cell proliferation at the interface in the presence of a dispersing mixture (Fig. 9), while the bacteria formed a dense biofilm in the sample containing hydrophobized halloysite after inoculation. The density estimate of *A. borkumensis* with a diameter of 20 microns increased five times from 0.3 to 1.5 bacteria per m^2^ (±20%, as estimated by direct calculation).

The density of *A. borkumensis* did not increase so dramatically within oil emulsions based on native halloysite. However, we observed the formation of microbubbles with a diameter of 2–3 μm on the surface of large droplets of oil (Fig. 9). The absence of bacterial cells at the interface in the case of a mixture of surfactants may be stipulated by the repulsion of negatively charged bacterial cells using anionic surfactant DOSS or lysis of bacterial cells under the action of surfactants. It was found that DOSS (also known as AOT) at a concentration of 1.8 mm (0.08 wt%) suppresses the growth of *A. borkumensis*.^74^ We used 1 wt% dispersing mixture containing 35 wt% DOSS and non-ionic surfactants, which resulted in a final DOSS concentration of 0.35 wt%. Similar growth was observed in samples of pure oil without halloysite. However, the bacterial mixture had to be well mixed in order to get a drop of emulsion small enough to observe the bacterial growth. In the case of an oil spill, dispersion of droplets with a diameter of 2 mm to 20 μm through halloysite Pickering stimulates a million times more surface area littered with hydrophobized halloysite, which contributes to bacterial prolongation emulsification.

## 8. Bioremediation technologies

Heavy metals are among the most dangerous pollutants and toxic elements for humans and the environment; among them, lead (Pb) and cadmium (Cd) are identified as the most negative ones. It has been noted that lead and cadmium are not decomposable and tend to accumulate in the food chain; therefore, for many countries, the issue of removing these elements from the environment is of paramount importance. Lactic acid bacteria (LAB) and lactobacilli in particular, have the ability to bind heavy metals, which makes them a promising tool for cleaning the environment and food industry products from heavy metals. Thus, based on the physicochemical properties of heavy metal elements, it is possible to specifically bind them with microorganisms, which proves to be an environmentally safe, inexpensive and effective method of lead and cadmium removal.^75^ A number of studies have shown that the accumulation of metal ions occurs on the cell surface due to physical adsorption,^76^ but there is no data on the accumulation of heavy metals in the cell during such adsorption.

The authors of this study have characterized the surface of microorganisms according to their potential ability to extract cadmium and lead from different liquids. In the research work, ten *Lactobacillus* strains, including four *L. plantarum* strains, three strains of *L. fermentum*, *L. brevis*, *L. buchneri* and *L. rhamnosus* have been investigated. Some of these strains were isolated from probiotics, dairy products and silage. Hydrophobic/hydrophilic surface properties of microbial cells, solvent adhesion and electrostatic properties of cell surface have been identified. In this work, the lowest investigated concentration of cadmium was 5 mg l^−1^ and it did not affect the growth of lactobacilli, with the exception of *L. fermentum* 3–2. This strain has shown decreased optical density values. The increase of cadmium concentration to 10 mg l^−1^ led to a decrease in optical density values for *L. plantarum* 8PA3, *L. plantarum* j-578, and *L. fermentum* 3–2, occurring in its stationary phase, as well as to complete growth inhibition in *L. brevis* 20054, *L. buchneri* 20057 and *L. rhamnosus* I2L. The highest concentration of Cd used in the experiments was 50 mg l^−1^. This concentration was toxic for all the studied samples. In the cultures of *L. plantarum* and *L. fermentum*, a significant reduction of growth has been revealed, whereas in the cultures of *L. brevis* 20054, *L. buchneri* 20057 and *L. rhamnosus* I2L, growth was inhibited for 18 hours. The addition of Pb in 5 mg l^−1^ and 10 mg l^−1^ concentrations for all the studied strains caused the inhibition of growth in microorganisms as compared to the control strain. However, the addition of lead in the above concentrations did not cause changes in the optical density values of *Lactobacilli*, except *L. plantarum* S1 (where the addition of lead has entailed an OD600 decrease by 14.5% and 30.4% for 5 mg l^−1^ and 10 mg l^−1^ of Pb, respectively). The highest test concentration for lead was also 50 mg l^−1^. The addition of this Pb concentration resulted in 100% growth inhibition for all lactobacilli. The study of microbial cells' external surface properties revealed that the most hydrophobic strains were *L. plantarum* B-578 (52.0 ± 6.4%), *L. brevis* 20054 (63.1 ± 5.6%) and *L. buchneri* 20057 (66.9 ± 6.3%). The lowest affinity for chloroform was observed in *L. fermentum* Na (9.8 ± 1.2%), and the highest affinity was observed in *L. plantarum* B-578 (88.8 ± 3.6%), *L. fermentum* 3–2 (93.8 ± 2.2%), *L. brevis* 20054 (94.6 ± 0.1%), and *L. buchneri* 20057 (97.1 ± 0.1%). The authors also measured the zeta potentials of microbial cells' surfaces. Surface charge in the studied strains was negative: the lowest for *L. plantarum* S1 was −34.9 ± 6.8 mV, and the highest charge was recorded for the strain of *L. fermentum* 3–3, which amounted to −7.4 ± 0.9 mV. All the measured values are presented in the table (Table 3). Importantly, the zeta potentials of the microbial cells' surfaces differed significantly between species and strains. After 1 hour of incubation in aqueous solutions containing 10 mg ml^−1^ of Cd or Pb, all strains of *Lactobacillus* showed a decrease in negative values of the cell surface zeta potential after incubation with Cd and Pb, but these changes were not statistically significant.

**Table 3:** Physicochemical properties of bacterial cell surface

Species and strains	Hexadecane	% of adhesion (±SD)* to ethyl acetate	Chloroform	*ζ* potential
***L. plantarum***				
8PA3	9.3 ± 2.2	34.5 ± 3.0	17.9 ± 1.6	−24.8 ± 2.5
B-578	52.0 ± 6.4	19.1 ± 2.0	88.8 ± 3.6	−27.6 ± 1.9
S1	6.6 ± 1.6	15.1 ± 2.3	29.3 ± 3.4	−34.9 ± 2.3
Ga	17.6 ± 3.8	14.6 ± 2.8	29.9 ± 2.6	−12.1 ± 1.2

***L. fermentum***				
Na	20.8 ± 3.1	18.4 ± 2.7	9.8 ± 1.2	−11.3 ± 0.7
3–2	27.9 ± 3.3	23.8 ± 2.2	93.8 ± 2.2	−19.3 ± 1.8
3–3	8.7 ± 1.8	10.7 ± 1.6	20.1 ± 2.1	−7.4 ± 0.9
*L. brevis* 20054	63.1 ± 5.6	26.5 ± 2.0	94.6 ± 0.1	−14.8 ± 1.8
*L. buchneri* 20057	66.9 ± 6.3	35.6 ± 3.1	97.1 ± 0.1	−20.7 ± 1.7
*L. rhamnosus* I2L	28.7 ± 3.3	21.3 ± 2.7	34.8 ± 4.0	−21.5 ± 2.2

The authors have also demonstrated that the studied *Lactobacillus* strains were resistant to Cd and Pb. The following strains proved to be the most susceptible to heavy metals: *L. brevis* 20054, *L. buchneri* 20057 and *L. rhamnosus* I2L, because they showed a significant decrease in growth when cadmium was added at a concentration from 10 mg l^−1^ to 50 mg l^−1^, whereas *L. fermentum* and *L. plantarum* strains continued to grow at these concentrations. Most microorganisms studied were relatively hydrophilic (Table 3), which is consistent with the reference sources data.^77^ Three strains of *L. plantarum* B-578 (52.0 ± 6.4%), *L. brevis* 20054 (63.1 ± 5.6%) and *L. buchneri* 20057 (66.9 ± 6.3%) were hydrophobic. The results of this study expand our the knowledge about the cell surface of lactobacilli and reveals the potential for the decontamination of Cd in four *L. plantarum* strains and three *L. fermentum* strains.

## 9. Conclusions

Clay nanoparticles are largely employed as adsorbent nanomaterials for pollutants because of their morphological characteristics, tuneable surface chemistry and sustainability. Their combination with organic molecules opens a new route in the fabrication of removal agents with targeted capacities towards aliphatic/aromatic hydrocarbons, organic dyes and heavy metals. Within this, hybrid gel beads formed by biopolymers (such as alginate and pectin) and halloysite nanotubes revealed as efficient systems for water decolouration, while the combination of kaolinite nanosheets and graphene oxides exhibited excellent remediation capacities. Supramolecular nanomaterials based on halloysite nanotubes and cucuriburil was successful in the toluene adsorption from both vapour and aqueous phase. The modification of halloysite cavity with anionic surfactants generated inorganic tubular micelles with a confined hydrophobic site efficient in the adsorption of aliphatic hydrocarbons including *n*-decane. Pickering emulsions based on halloysite nanotubes were tested for oil spill remediation, while novel bioremediation technologies were developed using lactic acid bacteria and *Lactobacilli*. Finally, clay nanomaterials are promising with respect to their reusability as they can be easily separated from water by simple sedimentation and adsorption/desorption phenomena can be reversed by controlling external parameters such as pH and ionic strength.

## Conflicts of interest

There are no conflicts to declare.

## Supplementary Material
